# Redox Imbalance Associates with Clinical Worsening in Spinocerebellar Ataxia Type 2

**DOI:** 10.1155/2021/9875639

**Published:** 2021-02-19

**Authors:** Almaguer-Gotay Dennis, Luis E. Almaguer-Mederos, Rodríguez-Aguilera Raúl, Rodríguez-Labrada Roberto, Velázquez-Pérez Luis, Cuello-Almarales Dany, González-Zaldívar Yanetza, Vázquez-Mojena Yaimeé, Estupiñán-Domínguez Annelié, Peña-Acosta Arnoy, Torres-Vega Reydenis

**Affiliations:** ^1^Center for the Investigation and Rehabilitation of Hereditary Ataxias (CIRAH), Holguín, Cuba; ^2^University of Medical Sciences of Holguín, Cuba; ^3^Cuban Academy of Sciences, Cuba

## Abstract

**Background:**

Spinocerebellar ataxia type 2 (SCA2) is a neurodegenerative disease presenting with redox imbalance. However, the nature and implications of redox imbalance in SCA2 physiopathology have not been fully understood.

**Objective:**

The objective of this study is to assess the redox imbalance and its association with disease severity in SCA2 mutation carriers.

**Methods:**

A case-control study was conducted involving molecularly confirmed SCA2 patients, presymptomatic individuals, and healthy controls. Several antioxidant parameters were assessed, including serum thiol concentration and the superoxide dismutase, catalase, and glutathione S-transferase enzymatic activities. Also, several prooxidant parameters were evaluated, including thiobarbituric acid-reactive species and protein carbonyl concentrations. Damage, protective, and OXY scores were computed. Clinical correlates were established.

**Results:**

Significant differences were found between comparison groups for redox markers, including protein carbonyl concentration (*F* = 3.30; *p* = 0.041), glutathione S-transferase activity (*F* = 4.88; *p* = 0.009), and damage (*F* = 3.20; *p* = 0.045), protection (*F* = 12.75; *p* < 0.001), and OXY (*F* = 7.29; *p* = 0.001) scores. Protein carbonyl concentration was positively correlated with CAG repeat length (*r* = 0.27; *p* = 0.022), while both protein carbonyl concentration (*r* = −0.27; *p* = 0.018) and OXY score (*r* = −0.25; *p* = 0.013) were inversely correlated to the disease duration. Increasing levels of antioxidants and decreasing levels of prooxidant parameters were associated with clinical worsening.

**Conclusions:**

There is a disruption of redox balance in SCA2 mutation carriers which depends on the disease stage. Besides, redox changes associate with markers of disease severity, suggesting a link between disruption of redox balance and SCA2 physiopathology.

## 1. Introduction

Spinocerebellar ataxias (SCAs) are a heterogeneous group of neurodegenerative diseases characterized by progressive neuronal loss and shared clinical manifestations, including gait ataxia, dysmetria, dysarthria, and adiadochokinesia [1]. To date, 48 molecular variants of SCAs have been reported [2]. In particular, spinocerebellar ataxia type 2 (SCA2) is due to a CAG repeat expansion mutation in the *ATXN2* gene and it reaches the highest worldwide prevalence in Holguín province, Cuba [3].

SCA2 is a polyglutamine disorder causing neurodegeneration at different levels, including cerebellar Purkinje cells, thalamic and cholinergic basal forebrain neurons, brainstem pontine, and olivary neurons, spinal and cortical motor neurons, as a result of mutant Ataxin-2 expression [4]. However, the primary mechanisms by which the polyQ expansion in Ataxin-2 causes SCA2 remain unknown. Nonetheless, evidence shows the occurrence of cytoplasmic aggregation of mutant Ataxin-2 [5], disturbed RNA metabolism [6, 7], dysregulation of calcium homeostasis [8, 9], altered methylation patterns in the *ATXN2* promoter [10], and oxidative stress as part of SCA2 physiopathology.

Oxidative stress was initially defined as “a disturbance in the prooxidant-antioxidant balance in favor of the former” and more recently as “a disruption of redox signaling and control” [11–14]. Enzymes like superoxide dismutase (SOD), catalase (CAT), and glutathione S-transferases (GSTs), or thiols as reduced glutathione (GSH), are part of the main antioxidant systems minimizing the damage caused by free radicals on lipids and proteins and regulate the cellular redox state [11, 12]. Oxidative stress has been linked to neurodegenerative disorders, including Alzheimer's disease (AD), Parkinson's disease (PD), amyotrophic lateral sclerosis (ALS), Huntington's disease (HD), and spinocerebellar ataxias [15–18].

Opposite to oxidative stress, the concept of “reductive stress” refers to a redox condition characterized by an abnormal increase in the levels of reducing agents in the forms of NADH, NADPH, and GSH and associated with increased mitochondrial oxidation and cytotoxicity [19]. Reductive stress has been poorly studied in the context of neurodegenerative disorders; however, evidence supporting its occurrence was gathered in young healthy individuals at risk of Alzheimer's disease [20] and in cellular and *Drosophila* models for Huntington's disease [21].

Few studies have shown the occurrence of redox imbalance in SCA2 patients. Indeed, increased levels of malondialdehyde (a product of lipids' oxidative damage), increased GST activity [22, 23], and decreased extracellular superoxide dismutase activity [24], were reported in SCA2 patients' blood serum. Besides, increased mitochondrial superoxide dismutase and decreased catalase expression were found in SCA2 patients' fibroblasts [25].

Even though evidence supporting the occurrence of redox imbalance in SCA2 was collected, its nature and implications in disease physiopathology have not been fully understood. Hence, the present study is aimed at assessing the redox imbalance and its association with disease severity in SCA2 mutation carriers by examining antioxidant and prooxidant parameters in a large sample of SCA2 patients and presymptomatic individuals.

## 2. Materials and Methods

### 2.1. Reagents

Pyrogallol (Alfa Aesar Co., Ward Hill, MA 01835, USA), hydrogen peroxide (H_2_O_2_) (Sigma-Aldrich, St. Louis, MO 63103, USA), 1-chloro-2,4-dinitrobenzene (Alfa Aesar, Ward Hill, MA 01835, USA), reduced glutathione (GSH) (Sigma-Aldrich, St. Louis, MO 63103, USA), 1,1,3,3-tetraethoxypropane (Sigma-Aldrich, St. Louis, MO 63103, USA), and bovine albumin (Calbiochem, San Diego, CA 92121, USA).

### 2.2. The Study Design

A case-control study was conducted to assess the redox status in SCA2 patients and presymptomatic individuals. Thirty-six molecularly confirmed SCA2 patients with mild to moderate clinical presentation (M/F: 4/32; aged 25 to 65 years) were recruited at the Center for the Investigation and Rehabilitation of Hereditary Ataxias, Holguín, Cuba. SCA2 patients were matched by age and gender with 36 presymptomatic individuals (mutation carriers with no clinical presentation at the time of the study) (M/F: 4/32; aged 23 to 66 years) and an equal number of healthy control individuals (M/F: 3/33; aged 23 to 65 years). To further verify differences between patients and controls regarding redox parameters, a data set consisting of 60 molecularly confirmed SCA2 patients (M/F: 27/33; aged 22 to 68 years) and 60 control individuals (M/F: 27/33; aged 23 to 70 years) matched by age and gender were assessed. A maximal age difference of two years between patients and presymptomatic and control individuals was allowed.

To assess the relationships between redox parameters, CAG repeat length, and clinical and neurophysiological variables, the sample of SCA2 patients was enlarged to one hundred (M/F: 48/52; aged 19 to 68 years). The study was approved by the institutional ethics committee, and it was conducted according to the Declaration of Helsinki. Written informed consent was obtained from all participants after a complete description of the study.

### 2.3. Clinical, Neurophysiological, and Genetic Assessment

The clinical diagnosis was based on the identification of gait ataxia, dysarthria, dysmetria, and dysdiadochokinesia and the slowing of saccade eye movements. Age at onset (AO) was defined as the onset of motor impairment. Disease duration (DD) was defined as the time elapsed between the clinical debut and the time when the neurological evaluation was made. Clinical severity was estimated by using the scale for the assessment and rating of ataxia (SARA) score [26]. The progression rate was calculated as the rate between the SARA score and age. Maximal saccade velocity (MSV) (in 60°/second) and saccade latency (SL) (in milliseconds) were determined as previously reported [27]. The mean estimated age at onset of the presymptomatic group was calculated with the individuals' current age and CAG repeat length at the *ATXN2* locus, as previously reported [28]. Predicted time to onset (in years) was calculated with the formula [predicted age of onset − current age]. The CAG repeat length at the *ATXN2* locus was determined by polymerase chain reaction (PCR) followed by polyacrylamide gel electrophoresis as previously reported [29].

### 2.4. Blood Sample Collection

Fasting blood samples were collected from subjects by venipuncture at the time when neurological and neurophysiological evaluations were made. Serum was obtained *via* blood centrifugation at 3,000 rpm at 4°C for 10 minutes, frozen immediately, and stored at −20°C until biochemical analysis.

### 2.5. Assessment of Antioxidant Biomarkers in Blood Serum

SOD3 and CAT enzymatic activities were measured at 37°C, following standard methods based on the use of pyrogallol and H_2_O_2_ as substrates [30, 31]. GST activity was measured at 37°C, following the Habig and Jacoby method, using 1-chloro-2,4-dinitrobenzene and GSH as substrates [32]. Reducing thiol (R-SH) total concentration was assessed at 25°C, following the Ellman protocol [33].

### 2.6. Assessment of Oxidative Modification on Lipids and Proteins

Thiobarbituric acid-reactive species (TBARS) and protein carbonyl (PC) concentration were assessed in blood serum following Yagi [34] and Levine [35] standard methods.

All samples were assayed in triplicate using a BioMate 3 Spectrophotometer (Thermo Spectronic Company, USA).

### 2.7. Redox Global Index Computation

The damage score (DS), protection score (PS), and OXY score were computed following a modified procedure to that reported by Veglia et al. [36]. The DS was computed with a base on log-transformed TBARS and PC concentrations; meanwhile, the PS was computed with a base on log-transformed SOD3, CAT, and GST enzymatic activities and R-SH concentrations. Individual redox parameters were standardized following Veglia et al. [36]. The OXY score was computed as the difference between DS and PS, reflecting the balance between oxidants and antioxidants.

### 2.8. Statistical Analysis

Descriptive statistics were used to assess central tendencies and dispersion of data. The Kolmogorov-Smirnov test was used to assess the normality of data distribution. The chi-square test (*χ*^2^) was used to establish comparisons between patients, presymptomatic individuals, and controls for gender. One-way ANOVA was utilized to assess differences between comparison groups for age and redox parameters. Tukey's post-hoc test was applied to identify differences between comparison groups. Student's *t*-test was used to compare redox parameters between patients and controls.

Pearson's correlation test was utilized to assess the relationship between redox parameters, clinical and neurophysiological variables, and CAG repeat length. Correction for repeat length or disease duration was applied to redox and clinical variables by simple or multiple linear regression analyses. Statistical significance was defined as *p* ≤ 0.05.

Type-I error in multiple comparisons was adjusted by the Benjamini-Hochberg (BH) method for controlling the false discovery rate [37]. Analyses were performed using the commercially available Statistica software package (StatSoft Inc., 2003 Statistica data analysis software system, version 6. http://www.statsoft.com).

## 3. Results

### 3.1. Redox Balance in SCA2 Mutation Carriers

To know if redox disturbances are present in SCA2 mutation carriers and to determine if these disturbances take place since the presymptomatic stage of the disease, comparisons for redox parameters were established between affected SCA2 patients and presymptomatic and control individuals. No significant differences were found between comparison groups for gender (*χ*^2^ = 0.20; *p* = 0.904) or age distributions (*F* = 0.656, *p* = 0.521). Also, no significant associations were found between age or gender and redox parameters in the comparison groups (*p* > 0.05).

There was no significant difference between SCA2 patients and presymptomatic and control individuals regarding SOD3 or CAT activities, SOD3/CAT index, R-SH, or TBARS concentrations. However, there were significant differences in GST activity, protein carbonyl concentration, damage score, and protective and OXY scores (Table 1). Nonetheless, only GST activity and protective and OXY scores remained significant after adjustment for multiple comparisons.

**Figure 1:**
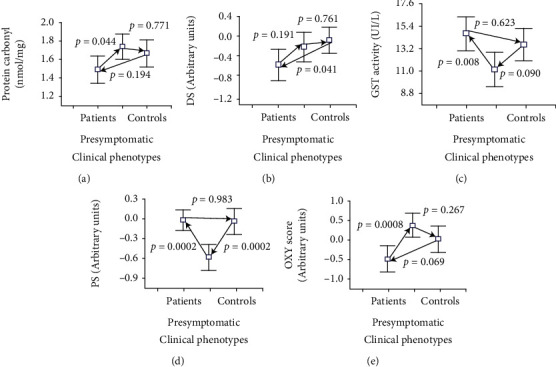
Post-hoc analyses for comparison of redox parameters between SCA2 mutation carriers and control individuals. DS: damage score; PS: protection score.

Post-hoc analyses showed significant decreases in GST activity and protection score and significant increases in protein carbonyl concentration and OXY score in presymptomatic individuals relative to affected patients. A significant decrease in the protection score was found in presymptomatic individuals relative to control individuals, and a significant decrease in the damage score was found in patients relative to control individuals (Figure 1). After correction for multiple comparisons, only the difference between presymptomatic individuals and affected patients relative to protein carbonyl concentration and the decrease in the damage score between patients and control individuals lost their statistical significance.

In the enlarged data consisting of 60 patients and 60 control subjects, significant decreases in the protein carbonyl concentration (*t* = −3.44; *p* < 0.001), damage score (*t* = −3.15; *p* = 0.002), and the OXY score (*t* = −2.55; *p* = 0.012) were found in patients relative to control individuals. These differences remained significant after adjustment for multiple comparisons.

### 3.2. Associations between Redox Parameters, CAG Repeat Length, and Clinical Biomarkers in SCA2 Mutation Carriers

Taking into account that the CAG repeat length in the *ATXN2* gene is the major determinant of clinical severity in SCA2, correlations were established between CAG repeat length and redox parameters in presymptomatic individuals and the enlarged sample of one hundred SCA2 patients. Besides, the relevance of redox parameters to clinical severity was assessed.

In presymptomatic individuals, a highly significant negative correlation between time to onset and repeat length (*r* = −0.48; *p* = 0.005) was found. However, no significant correlation was obtained between repeat length and redox parameters (*p* > 0.05). As expected, in the enlarged sample of one hundred SCA2 patients, the age at onset showed a highly significant negative correlation with the repeat length (*r* = −0.73; *p* < 0.001), and the SARA score was significantly correlated to the disease duration (*r* = 0.57; *p* < 0.001) and repeat length (*r* = 0.21; *p* = 0.038). Also, the progression rate showed significant correlations with repeat length (*r* = 0.71; *p* < 0.001) and disease duration (*r* = 0.23; *p* = 0.022). Besides, saccade velocity was significantly correlated to repeat length (*r* = −0.46; *p* < 0.001). Nonetheless, saccade latency showed no significant correlations with repeat length or disease duration.

**Figure 2:**
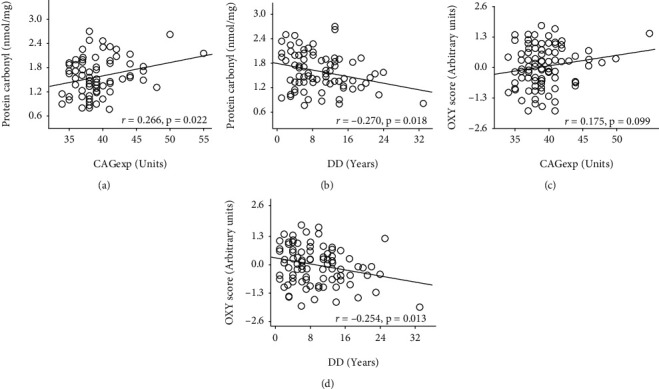
Correlation analyses for redox parameters, repeat length, and disease duration. CAGexp: CAG repeat length in ATXN2 expanded alleles; DD: disease duration.

On correlation analysis, the repeat length showed a significant association only with protein carbonyl concentration. Similarly, the disease duration showed significant negative correlations with protein carbonyl concentrations and OXY score (Figure 2).

Significant effects were obtained for the repeat length (*β* = 0.283, SE = 0.11; *p* = 0.012) and disease duration (*β* = −0.280, SE = 0.11; *p* = 0.014) on protein carbonyl concentration by multiple linear regression analysis (*R* = 0.387; *p* = 0.003). Besides, significant effects were obtained for the repeat length (*β* = 0.268, SE = 0.10; *p* = 0.009) and disease duration (*β* = −0.240, SE = 0.10; *p* = 0.019) on the OXY score (*R* = 0.357; *p* = 0.003).

Regarding the associations between redox parameters and clinical biomarkers, the time to onset in presymptomatic individuals showed significant correlations with R-SH (*r* = 0.41; *p* = 0.025) and protein carbonyl concentration (*r* = 0.35; *p* = 0.049). However, after correction for repeat length, only the association with protein carbonyl concentration remained significant. Also, a correlation of marginal significance was obtained for the damage score (Table 2).

In affected patients, there was no significant correlation between the age at onset and redox parameters. Nonetheless, after correction for repeat length, the age at onset showed significant negative correlations with R-SH concentration and the protection score. Corrected age at onset also showed significant positive correlations with protein carbonyl concentration and the OXY score (Table 2).

The SARA score showed significant correlations with GST activity (*r* = 0.22; *p* = 0.036), CAT activity (*r* = 0.29; *p* = 0.004), CAT/SOD3 index score (*r* = 0.29; *p* = 0.006), protein carbonyl concentration (*r* = −0.30; *p* = 0.01), damage score (*r* = −0.27; *p* = 0.008), protection score (*r* = 0.31; *p* = 0.002), and OXY score (*r* = −0.39; *p* < 0.001). After correction for disease duration and repeat length, the SARA score showed significant positive correlations with GST and CAT activities, the CAT/SOD3 index score, and the protection score.

Likewise, corrected the SARA score showed significant negative correlations with the damage and OXY scores (Table 2). Similarly, the progression rate showed significant correlations with CAT (*r* = 0.25; *p* = 0.015) activity and the CAT/SOD3 index score (*r* = 0.32; *p* = 0.002). After correction for repeat length and disease duration, the progression rate was positively correlated to CAT activity, the CAT/SOD3 index score, and the protection score. Besides, the corrected progression rate was negatively correlated to protein carbonyl concentration and the damage and OXY scores (Table 2). On the other hand, saccade velocity showed significant correlations with CAT activity (*r* = −0.35; *p* = 0.008) and the CAT/SOD3 index score (*r* = −0.33; *p* = 0.013). After correction for repeat length, saccade velocity showed a highly significant negative correlation with CAT activity. On the contrary, saccade latency showed a significant positive correlation with CAT activity (Table 2).

## 4. Discussion

In this study, taking advantage of the largest and genetically homogeneous SCA2 population worldwide, the evidence is provided for the role of oxidative stress in the presymptomatic stage of the disease, which seems to evolve into reductive stress in symptomatic stages, then contributing to clinical worsening. To our knowledge, this is the first study on redox balance in SCA2 which includes presymptomatic individuals, showing a redox shift in the transition from presymptomatic to symptomatic stages of the disease.

Presymptomatic individuals presented a significantly higher protein carbonyl concentration and OXY score, in parallel to lower GST activity than affected patients. Besides, presymptomatic individuals presented significantly lower protection scores than affected patients and healthy controls. Overall, this evidence indicates the occurrence of oxidative stress in the presymptomatic stage of the disease, which seems to be harmful as the corrected protein carbonyl concentration is negatively associated with the time to disease onset. This finding suggests that protein carbonyl concentration might be a good predictor of disease onset in mutation carriers.

As far as we know, only two studies have assessed the relevance of redox parameters in presymptomatic individuals for polyglutamine disorders. In presymptomatic individuals for Huntington's disease, higher levels of lipid peroxidation and protein carbonyl concentration and lower GSH concentration were found, suggesting the occurrence of oxidative stress before the onset of HD symptoms [38]. Also similar to our findings in SCA2, no significant associations were found for repeat length and redox parameters in HD presymptomatic individuals [38].

Contrary to our results in SCA2 presymptomatic individuals, higher SOD3 and glutathione peroxidase activities and decreased levels of reactive oxygen species were found in presymptomatic individuals with spinocerebellar ataxia type 3 (SCA3), suggesting a potential antioxidant adaptive response to an oxidative challenge taking place before disease onset. Nonetheless, these results seem to be of limited pathological significance as no correlations of redox markers with the predicted age of onset or repeat length were found [18].

Though oxidative stress has been suggested to be playing key roles in neurodegenerative disorders, this oxidative stress-centered view was challenged by evidence of little or no protection by free radical-scavenging antioxidants [39–41] and by evidences showing no association between clinical progressions and the oxidation induced by free radicals in the brain [42, 43]. In addition, findings relative to increased glucose 6-phosphate dehydrogenase (G6PDH) in AD and PD [44, 45] and increased thioredoxin reductase (TrxR) [46], neuronal thiols, and GSH/GSSG ratio in AD [44, 47] suggest that reductive reprogramming has an important role in these disorders. Also, the occurrence of lower GSSG and P-p38 levels and higher expression of glutamylcysteinyl ligase and glutathione peroxidase in young healthy individuals at risk of Alzheimer's disease [20] and the enhancing of the neurodegenerative phenotype in cellular and *Drosophila* models for Huntington's disease by treatment with N-acetyl-L-cysteine and overexpression of SOD1 support a relevant role for reductive stress in these neurodegenerative disorders [21].

In this study, it was found that affected SCA2 patients show a significant decrease in the protein carbonyl concentration and damage and OXY scores relative to controls, probably as a result of increased antioxidant activity. In addition, longer CAG repeats were associated to increased protein carbonyl concentration, as a reflection of the damaging effects of mutant Ataxin-2. Unexpectedly, there was a decrease in protein carbonyl concentration and OXY score with the advance of the disease, suggesting the sustained activation of the antioxidant machinery. Indeed, increased GST activity was observed in our patients and increased concentration of GSH was found in the white matter of SCA2 patients in a previous report [48].

Similar increases in the antioxidant defenses have been traditionally interpreted as an adaptive response to counteract the damaging effects of prooxidants on the cellular major macromolecular components, with a global protective effect [15, 16, 49]. However, we found that SCA2 patients with early disease onset show higher reducing thiol total concentration and protection score, suggesting harmful effects for increased antioxidant defenses. Moreover, positive associations were found for antioxidant parameters and SARA score, progression rate, and saccade latency, as well as a negative association with saccade velocity, reinforcing the suggestion that increased antioxidant defenses have worsening effects on the clinical presentation. In particular, catalase activity seems to be of great relevance as it was associated with the SARA score, disease progression, saccade velocity, and latency. In addition, SCA2 patients with early disease onset show lower protein carbonyl concentration and there were inverse significant associations between prooxidant parameters and disease severity.

Overall, this evidence suggests the potential occurrence of reductive stress in the symptomatic stage of SCA2, which reinforces the neurodegenerative process probably by contributing to the loss of ROS physiological effects on normal neuronal development and function.

It has been shown that ROS are important in the establishment of neuronal polarity and growth cone pathfinding [50, 51] and in the regulation of synaptic transmission and plasticity [52, 53]. Indeed, superoxide is essential to induce long-term depression in the cerebellar Purkinje neurons [54] and physiological concentrations of hydrogen peroxide are necessary for structural plasticity dependent on neuronal activity and for the maintenance of evoked synaptic transmission in *Drosophila* [55]. On the contrary, activation of catalase inhibited the adaptive morphological changes of the neuromuscular junction in *Drosophila* [55]. Besides, overexpression of mitochondrial catalase, producing an astrocyte-specific decrease of endogenous mitochondrial ROS in mice, causes profound changes in brain energy and redox metabolism, eventually leading to neuronal dysfunction and cognitive impairment [56]. This evidence suggests that overactivation of the antioxidant machinery might have negative effects on the physiology of the nervous system.

It has been proved that neuronal polarization depends heavily on the activity of protein kinases including PI3K, whose signaling can be regulated by ROS-mediated inhibition of PTEN [57]. PTEN and PI3K were also involved in synaptic terminal growth by DJ-1*β* oxidation in *Drosophila* [58, 59]. In addition, the release of calcium from intracellular stores was involved in the mechanisms by which ROS contribute to neuronal polarity and to synaptic plasticity [54, 60, 61]. Importantly, Ataxin-2 protein was implicated in calcium signaling and PI3K/Akt/mTOR pathways [8, 9, 62]. These findings provide potential links between Ataxin-2 and redox-mediated neuronal development and function.

In conclusion, there is a disruption of redox balance in SCA2 mutation carriers which depends on the disease stage. Besides, redox changes associate with markers of disease severity, suggesting a link between disruption of redox balance and SCA2 physiopathology. Further studies are needed to confirm these findings, to clarify the molecular mechanisms involved and to assess the usefulness of redox parameters as biomarkers of disease progression and to monitor the effects of therapeutic interventions.

## Figures and Tables

**Table 1:** Comparison of redox parameters between SCA2 mutation carriers and control individuals.

Redox parameters	Controls (*n* = 36)		Presymptomatics (*n* = 36)		Patients (*n* = 36)		*F*	*p*
	Mean	SD	Mean	SD	Mean	SD		
SOD3 (KU/L)	2.02	0.52	1.85	0.46	1.99	0.38	1.46	0.238
CAT (KUI/L)	19.14	8.74	15.48	7.68	20.00	13.81	1.91	0.153
CAT/SOD3	9.97	5.46	9.30	6.39	10.35	6.86	0.26	0.773
GST (UI/L)	14.00	4.73	11.54	5.08	15.09	4.98	4.88	*0.009*
R-SH (*μ*mol/L)	136.92	71.29	105.09	43.30	124.32	63.17	2.54	0.084
TBARS (*μ*mol/L)	2.17	0.72	1.90	0.72	1.97	1.07	0.88	0.420
PC (nmol/mg)	1.65	0.43	1.74	0.41	1.48	0.43	3.30	*0.041*
DS	0.03	0.74	−0.11	0.83	−0.44	0.86	3.20	*0.045*
PS	−0.01	0.51	−0.49	0.50	0.01	0.41	12.75	*<0.001*
OXY score	0.04	0.97	0.38	0.87	−0.46	0.97	7.29	*0.001*

SOD3: superoxide dismutase activity; CAT: catalase activity; GST: glutathione S-transferase activity; R-SH: reducing thiol total concentration; TBARS: thiobarbituric acid-reactive species; PC: protein carbonyl concentration; DS: damage score; PS: protection score; SD: standard deviation.

**Table 2:** Correlation analyses for redox and clinical parameters.

Redox/clinical parameters	Correlation coefficient (*p* level)					
	TTO^♦^	AO^♦^	SARA score^◊^	PR^◊^	Sac. veloc.^♦^	Sac. lat.
SOD3	−0.04 (0.811)	−0.04 (0.714)	0.09 (0.428)	0.10 (0.358)	−0.031 (0.824)	0.15 (0.294)
CAT	−0.06 (0.751)	−0.07 (0.504)	0.44 (*<0.001*)	0.50 (*<0.001*)	-0.31 (*0.025*)	0.31 (*0.022*)
CAT/SOD3	0.08 (0.650)	−0.06 (0.574)	0.34 (*0.002*)	0.38 (*<0.001*)	−0.21 (0.127)	0.14 (0.303)
GST	−0.03 (0.885)	−0.09 (0.407)	0.25 (*0.017*)	0.21 (0.051)	0.17 (0.221)	−0.16 (0.249)
R-SH	0.28 (0.131)	−0.23 (*0.027*)	0.07 (0.493)	0.17 (0.112)	−0.26 (0.060)	0.21 (0.122)
TBARS	−0.18 (0.347)	0.07 (0.510)	−0.19 (0.083)	−0.16 (0.16)	−0.05 (0.742)	0.06 (0.653)
PC	−0.43 (*0.014*)	0.29 (*0.013*)	−0.20 (0.094)	−0.25 (*0.033*)	−0.04 (0.799)	0.006 (0.971)
DS	−0.34 (0.058)	0.18 (0.084)	−0.24 (*0.025*)	−0.22 (*0.036*)	−0.04 (0.782)	−0.02 (0.880)
PS	−0.05 (0.774)	−0.30 (*0.004*)	0.35 (*0.001*)	0.44 (*<0.001*)	−0.21 (0.134)	0.25 (0.072)
OXY score	−0.29 (0.111)	0.30 (*0.005*)	−0.38 (*<0.001*)	−0.41 (*<0.001*)	0.09 (0.528)	−0.15 (0.261)

SOD3: superoxide dismutase activity; CAT: catalase activity; GST: glutathione S-transferase activity; R-SH: reducing thiol total concentration; TBARS: thiobarbituric acid-reactive species; PC: protein carbonyl concentration; DS: damage score; PS: protection score; SD: standard deviation; TTO: time to onset (in presymptomatic individuals); AO: age at onset; PR: progression rate. ^♦^Corrected for repeat length; ^◊^corrected for disease duration and CAG repeat length.

## Data Availability

The molecular and clinical data used to support the findings of this study are restricted by the Ethics Committee at the Center for the Investigation and Rehabilitation of Hereditary Ataxias in order to protect patient privacy. Data are available from D Almaguer-Gotay for researchers who meet the criteria for access to confidential data.
